# Automated plate-to-peptide protocol for mass spectrometry sample preparation

**DOI:** 10.1016/j.xpro.2026.104917

**Published:** 2026-10-28

**Authors:** Sibilla Sander, Sophie Buchser, Michael Stumpe, Joern Dengjel

**Affiliations:** 1 Department of Biology, University of Fribourg, Chemin Du Musée 10, 1700 Fribourg, Switzerland

**Keywords:** Cell culture, Cell separation/fractionation, Proteomics

## Abstract

Although advances in mass spectrometry now support proteomic screens of thousands of samples and sample preparation has been automated to match, most current protocols still start from pre-prepared cell lysates. Here, we present a protocol for automated mass spectrometry sample preparation starting at the cell culture plate level. We describe steps for cell seeding in 96-well plates, in-plate cell lysis, and single-pot solid-phase-enhanced sample preparation. This streamlined approach significantly reduces manual handling while yielding highly reproducible measurements from low cell numbers.

## Highlights

• Instructions for cell seeding and lysis in 96-well cell culture plates
• Instructions for preparation of the Bravo liquid handling platform
• Steps for mass spectrometry sample preparation in 96-well cell culture plates

## Abstract (editor highlights)

Publisher’s note: Undertaking any experimental protocol requires adherence to local institutional guidelines for laboratory safety and ethics.

## Graphical abstract

**Figure:**
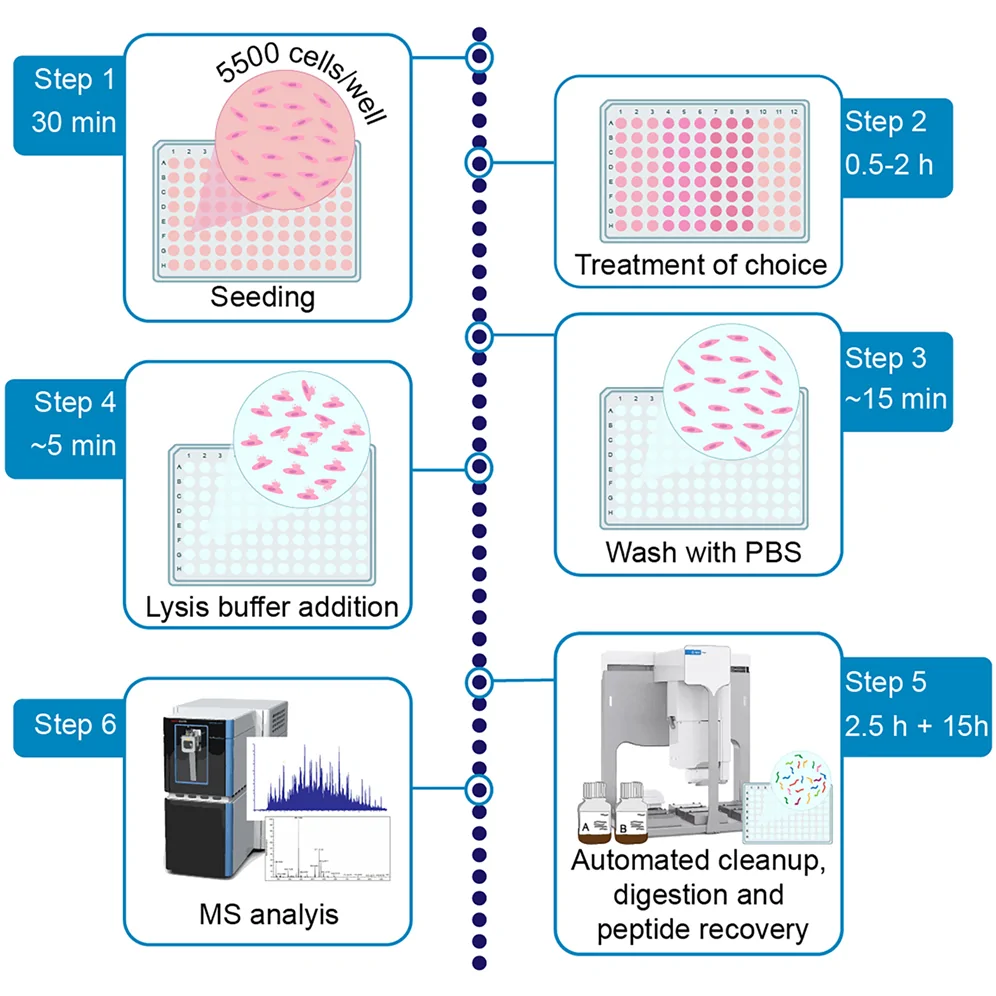

## Before you begin

Mass spectrometry (MS)-based proteomic technologies quickly evolve, gaining speed and sensitivity.^1^ Especially the introduction of ion mobility as an additional dimension to separate and store ions,^2^ and new ultra-fast and sensitive mass analyzers with single-ion sensitivity,^3^ in combination with data-independent acquisition and new data analysis software,^4^ substantially increase data completeness and sample throughput.^1^ In this context, automation in sample preparation is critical to support the higher throughput and increase reproducibility. Different versions of sample preparation approaches relying on the capturing of proteins on (para)magnetic beads, such as single-pot solid-phase-enhanced sample preparation (SP3) and protein aggregation capture (PAC),^5^^,^^6^ are extremely popular, as samples are prepared in a 96-well plate format. As these workflows are compatible with a variety of buffer systems, they contribute to robust peptide identification by MS.^7^ Automation starts at the level of cell lysates, i.e., protein extracts. Here, we present the plate-to-peptide (P2P) workflow, a SP3-based protocol designed for automated sample preparation starting at the cell culture plate level. The protocol outlines a low-input, high-throughput workflow starting from 96-well adherent cell culture plates.

### Innovation

This protocol outlines a high-throughput automated P2P sample preparation workflow for MS analysis. In contrast to existing low-input proteomic workflows that require early transfer of cell lysates, specialized consumables, or MS-compatible detergents, this method combines SDS-based lysis with SP3-based digestion within the original cell culture wells prior to peptide recovery. Automation on the Agilent Bravo liquid handling platform reduces sample loss and well-to-well variability, which is critical for low cell numbers.

Using this approach, robust data-independent acquisition (DIA)-based proteomic profiles can be obtained from approximately 5,500 adherent cells per well, yielding reproducible quantification of more than 8,000 proteins on state-of-the-art mass spectrometers while maintaining compatibility with high-throughput experimental designs, such as drug screening,^8^ or characterization of cell populations.^9^

By combining cell culture, lysis, cleanup, and digestion in the same plate, this protocol supports the analysis of cell-secreted extracellular matrix (ECM) and allows a scalable solution for automated proteomics in high-throughput screens and other applications requiring minimal manual handling. This workflow is adapted from Müller et al.^5^ and Sander et al.^10^ starting from 96-well cell cultures plates.

### Institutional permissions (if applicable)

Ethical approval should be obtained in the early stages of the planning process and must be obtained before any data collection from primary human cells.

### Seeding cells

**Timing: ∼1 h**

Seed cells in Dulbecco’s Modified Eagle’s Medium, supplemented with 10% fetal bovine serum and 100 units/ml penicillin and 0.1 mg/ml streptomycin one day before the experiment.

***Note:*** Different cell types can be used, and the number of cells used should be adjusted to ensure the desired confluence.

1. Remove medium from cell culture dishes by aspiration.
2. Wash cells twice with sterile Dulbecco’s Phosphate-Buffered Saline (DPBS).
3. Add enough trypsin-EDTA to cover the cells.
4. Place them at 37°C until the cells detach from the plate, commonly 5 min.
5. Resuspend the cells in complete medium to stop the trypsin-EDTA reaction.
6. Collect all the suspension in a 15 ml tube.
7. Determine cell concentration using a cell counter.

***Note:*** Cell concentration can also be determined by manual counting.

8. Dilute the cells to a final concentration of 55000 cells/ml.
9. Distribute 100 μl of cell suspension per well in a 96-well plate (Troubleshooting 1).

### Adding new labware to the VWorks automation control software of the Bravo liquid handling platform

10. Open the labware editor (“Tools” > “Labware Editor…”).
11. Select “96 Corning 9017, Flat bottom, polystyrene, clear.”
12. Press “Save changes as…”
13. Modify the name to “96 VWR 734–2327, Flat bottom, polystyrene, clear.”

**CRITICAL:** Do not change this name. The name needs to correspond to the one present in the form file, or it will not be found, and the protocol will fail to work.

14. Set the parameters as shown in Figure 1 (Troubleshooting 2).

**Figure 1:**
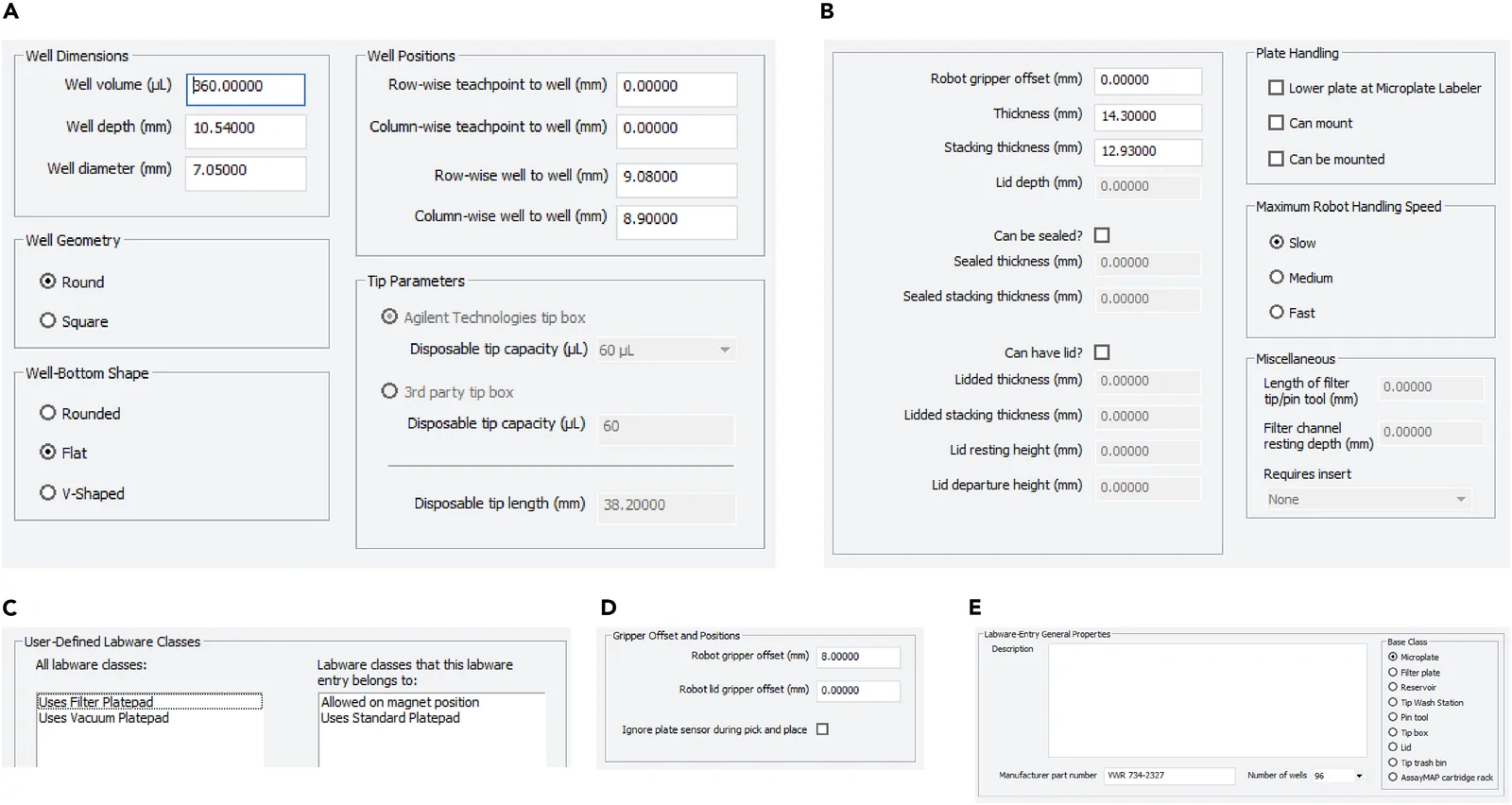
Settings for new labware addition to VWorks (A) Pipette/Well Definition tab in the Labware Editor. (B) Plate properties tab. (C) Labware Classes tab. (D) Bravo tab. (E) Labware Entry General Properties.

## Key resources table

**Table:** 

REAGENT or RESOURCE	SOURCE	IDENTIFIER
**Chemicals, peptides, and recombinant proteins**		

Dulbecco’s Modified Eagle Medium (DMEM) high glucose 4.5 g/L, stable glutamine, with sodium pyruvate	PAN Biotech	P04-04510
Dulbecco’s Phosphate-Buffered Saline 1× w/o Ca and Mg	PAN Biotech	P04-36500
Fetal Bovine Serum (FBS)	BioWest	S181B-500
Trypsin 0.05%-EDTA 0.02% in DPBS solution	PAN Biotech	P10-023100
Penicillin-Streptomycin	PAN Biotech	P06-07100
EDTA disodium salt (Ethylene Diamine Tetra Acetate disodium salt)	Sigma	E5134
Sodium dodecyl sulfate (SDS)	Sigma	L5750
NP-40 Alternative	Millipore	492016
Sodium chloride (NaCl)	Roth	3957.4
Trifluoroacetic acid (TFA)	Sigma-Aldrich	302031
MS-grade Water	VWR	23595.328
MS-grade Acetonitrile	VWR	20060.32
Ethyl alcohol, pure, ≥99.9% (GC), gradient grade	Supelco	1117272500
Benzonase	Dr. Nuclease Benzonase	BP4200
Sequencing grade modified trypsin	Promega	V5113
Dithiothreitol	VWR	0281
Iodoacetamide	Sigma	4386
Water with 0.1 % Formic Acid (v/v), Optima LC/MS grade	Fisher Scientific	LS118
Acetonitrile with 0.1 % Formic Acid (v/v), Optima LC/MS grade	Fisher Scientific	LS120
Ammonium bicarbonate	Sigma	9830
Sera-Mag™ Speedbeads	GE Healthcare	45152105050250
Sera-Mag™ Speedbeads	GE Healthcare	65152105050250
Acetic acid 99% ≥99%, HiPerSolv CHROMANORM® for LC-MS	VWR	84874.26

**Critical commercial assays**		

Quantitative colorimetric Peptide Assay kit	Fisher scientific	23275

**Software and algorithms**		

Spectronaut	Biognosys	N/A
VWorks Automation Control Software	Agilent	installer 13.1.12.1543
P2P workflow files for VWorks	Agilent	Version 1.0.0

**Other**		

Bravo liquid handling platform (Bravo)	Agilent	RRID:SCR_026137
Orbitrap Astral mass spectrometer	Thermo Fisher Scientific	RRID:SCR_026205
Vanquish Neo UHPLC system	Thermo Fisher Scientific	RRID:SCR_026495
Greiner Bio-One 96-well microplate for PCR, skirted	Greiner	652270B
NanoDrop One Microvolume UV-Vis Spectrophotometer	Thermo Scientific	ND-ONE-W
Tissue Culture plates 96-wells-F, surface treated, sterile	VWR Europe	734-2327
96-well magnet	Alpaqua	A000400
Reservoir, 6 column, polypropylene, 47 ml/column	Agilent	201284-100
250 μl tips boxes	Agilent	19477-002
Adapter heating station, Flat Bottom Plate, Inheco	Agilent	G5550-20070
Multichannel pipette	N/A	N/A
Eppendorf ThermoMixer® C	Eppendorf	5382000015
ThermoTop®	Eppendorf	5308000003
Eppendorf SmartBlock plates	Eppendorf	5363000039
Sealing tape for 96-well plates, aluminum, non-sterile	Corning	6570
Orbital shaking station	Agilent	G5431B
Bravo 96 channel disposable tip head LT	Agilent	G5055A
Magnet for 1.5–2 ml tubes	N/A	N/A
1.5 ml microcentrifuge tubes	N/A	N/A

## Materials and equipment

**Table:** Lysis buffer

Reagent	Final concentration
Tris pH 7.5	50 mM
NaCl	150 mM
EDTA	1 mM
NP-40	1%
SDS	1%

Storage at 20–25°C for up to one month. Add benzonase (1:1000) before use.

**Table:** Dithiothreitol (DTT) stock solution

Reagent	Final concentration
DTT	100 mM
ddH_2_O	–

Storage at −20°C for up to three months. To be diluted in water to 3 mM before use.

**Table:** Iodoacetamide (IAA) stock solution

Reagent	Final concentration
IAA	550 mM
ddH_2_O	–

Storage at −20°C in the dark for up to three months. To be diluted in water to 22 mM before use.

**Table:** Ammonium bicarbonate (ABC) buffer

Reagent	Final concentration
Ammonium bicarbonate	100 mM
ddH_2_O	–

Storage at 20–25°C for up to one month.

**Table:** EtOH 80%

Reagent	Final concentration
Ethanol	80% v/v
ddH_2_O	–

Storage at 20–25°C for up to one month.

**Table:** TFA 5%

Reagent	Final concentration
Trifluoroacetic acid	5% v/v
ddH_2_O	–

Storage at 20–25°C for up to one month.

**Table:** Acetic acid solution

Reagent	Final concentration
Acetic acid	50 mM
ddH_2_O	–

Storage at 20–25°C for up to one month.

### SP3 bead suspension

• Resuspend the stock solutions well by gentle shaking (Figure 2A).

**Figure 2:**
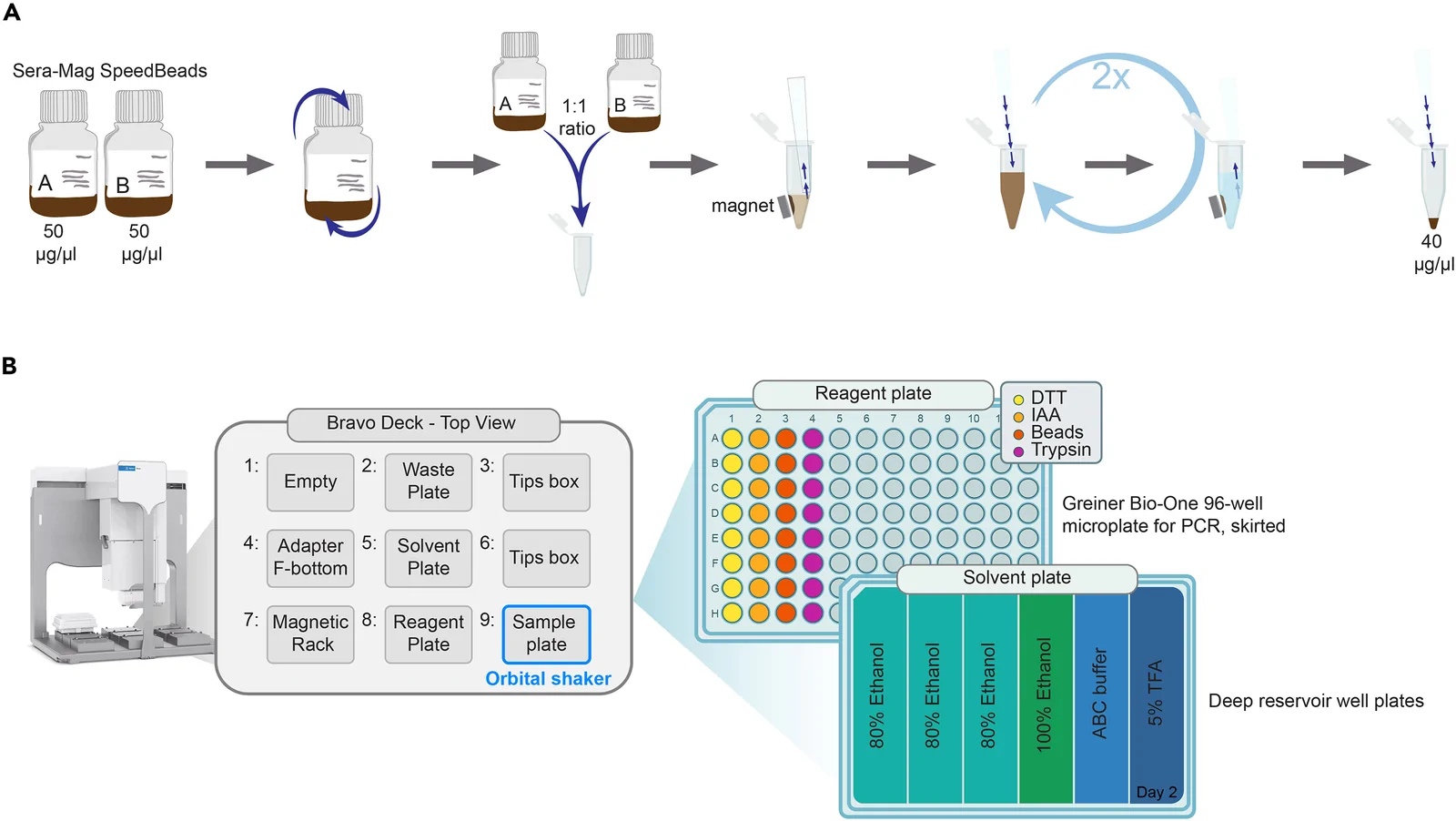
Setup for P2P protocol (A) The concentration of each stock solution of SeraMag SpeedBeads is 50 μg/μl. Before use, they should be carefully resuspended and combined into a reaction tube at a ratio of 1:1, washed twice, and finally resuspended at a concentration of 40 μg/μl in ddH_2_O. (B) Reagents and solvents plates should be positioned respectively in positions 8 and 5. Adapted from.^10^
• Mix 252.8 μl of each bead stock solution in a microcentrifuge tube (GE Healthcare, Cat. No. 65152105050250 and Cat. No. 45152105050250 - stock concentrations 50 μg/μl).
• With the help of a magnet, wash twice with 1 ml of ddH_2_O.
• Resuspend in 632 μl of ddH_2_O (final concentration 40 μg/μl).

This is the amount of SP3 beads suspension for a full 96-well plate. It can be stored at 4°C for 1 month.

### Workflow files on Bravo liquid handling platform

We adapted the autoSP3 workflow version 1.0.4 from Agilent.^5^

Before using this workflow, make sure to have the magnet in position 7, the heating plate with flat-bottom plate adaptor in position 4, and the orbital shaker in position 9 (Position 1 is the wash station, and it is not used in this workflow) of the Agilent Bravo working deck, as shown in Figure 2B.

The modified files can be obtained from Agilent free of charge. The Agilent Automation team can be contacted to support the installation.

## Step-by-step method details

### Preparation of the Bravo liquid handling platform

**Timing: 15 min**

This step includes the preparation of the robot for clean-up and trypsin addition for a complete 96-well plate.

1. Open the protocol.

***Note:*** Opening the protocol file will also open the form file and prompt the initialization of the robot. If not prompted, open the device file.

2. Initialize the robot.
  a. Ignore and continue homing the G-axis.
  b. Retry homing the W-axis.

**CRITICAL:** The initialization is essential. Without it, the robot will not be correctly configured for the protocol.

3. In the tab load/save method, load the P2P method.
4. In the input parameter tab (Figure 3A):
  a. Perform lysis and reduction.
  b. Perform alkylation.
  c. Perform beads + EtOH addition.
  d. Perform beads EtOH removal (starts from loc 7).
  e. Perform solvent wash.
  f. Perform ABC + trypsin resuspension.

**Figure 3:**
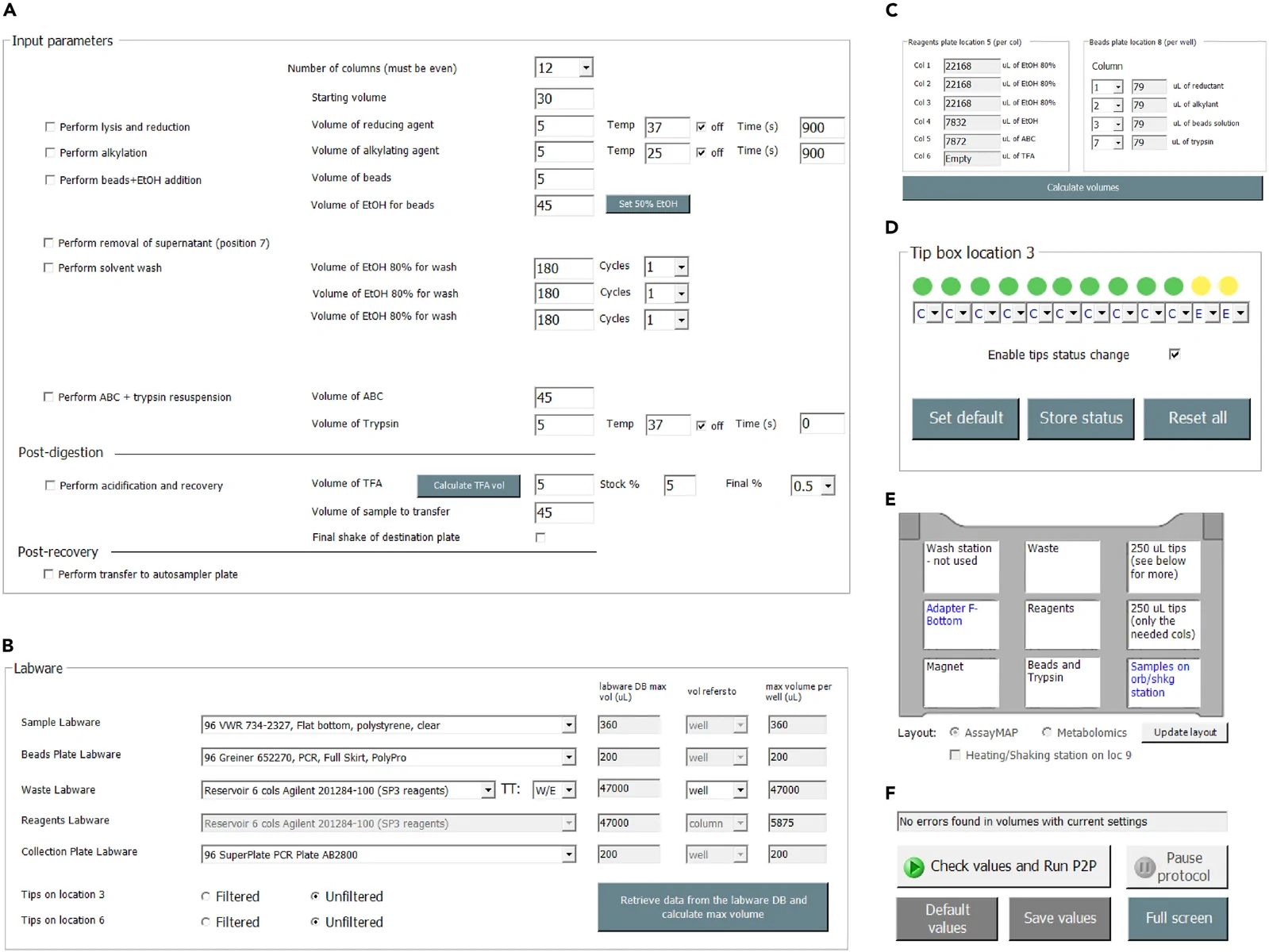
Main panels of the interface in VWorks for P2P protocol (A) For the current workflow, the number of columns on the sample plate should be set to 12. The ticked checkboxes with the associated volumes are the ones necessary for day one of the workflow. Every step can be adapted by the user. (B) Dropdown menus allow the selection of the specific labware. The labware shown in the image is the one used for the workflow. (C) Volume needed for the reagent and solvent plates. By pressing “calculate volume” the form will define the volume of each reagent based on the chosen steps. If the entered values are out of range, an error message will be displayed. (D) Default setup of the tips in position 3. (E) The layout “AssayMAP” should be selected for this workflow. (F) By pressing Check values and Run P2P the workflow will start and the program will check the values. In case of problems, an error message will be displayed.
5. In the labware, reagent and tips tab, press “Retrieve data from labware DB and calculate max volume” and “calculate volumes” (Figure 3 - Troubleshooting 3).
6. Prepare the reagent plate in a V-bottom 96-well plate.
  a. Add 79 μl of 3 mM DTT in each well of the first column.
  b. Add 79 μl of 22 mM IAA in each well of the second column.
  c. Add 79 μl of bead suspension in each well of the third column (Troubleshooting 4).
  d. Add 79 μl of 0.08 μg/μl trypsin in 50 mM acetic acid to each well of the fourth column.

***Note:*** The columns of the reagent plate can be changed in the program.

7. Prepare the solvent plate in a 6-column reservoir plate as shown in Figure 1B.
  a. Add 22.2 ml of 80% EtOH to each of the first 3 columns.
  b. Add 7.9 ml of 100% EtOH to the fourth column.
  c. Add 7.9 ml of ABC buffer to the fifth column.
8. Prepare the robot as shown in Figure 2B.
  a. Place a waste 6-column deep reservoir plate in position 2.
  b. Place a box of tips without the last two columns in position 3.
  c. Place the solvent plate in position 5.
  d. Place a full box of tips in position 6.
  e. Place the reagent plate in position 8.
9. Set the tips information for position 3 in the labware, reagent, and tips tab (Figure 3D).
  a. Tick “Enable tips status”.
  b. Press “Set default”.
10. Select the layout “AssayMAP” (Figure 3E).
11. Press “Update layout“.

### Washing and lysis of the cells in the plate

**Timing: ∼20 min**

This is a manual step, and it includes the harvesting of the cells and the addition of lysis buffer to the cell culture 96-well plate. Cells can be harvested in advance, and once the PBS is removed, the dry plate can be stored at −80°C.

12. Fill a reservoir with DPBS and place it on ice.
13. Place the 96-well plate on ice.
14. Aspirate the medium carefully.

***Note:*** Use smaller tips and aspirate at the edge of the well to avoid cell loss.

15. Wash twice with 100 μl ice-cold DPBS.
16. Remove the DPBS completely.
  a. Hold the plate with an inclination.
  b. Remove the DPBS by careful aspiration.

**CRITICAL:** Leftover DPBS will dilute the lysis buffer. Gel-loading pipette tips can be used as aspiration tips to be more precise.

**Pause point:** The plate can be sealed with sealing tape for 96-well plates and stored at −80°C.

17. Add 30 μl of lysis buffer to each well using a multichannel pipette.

### Plate-to-peptide SP3-based sample cleanup and digestion

**Timing: 2 + 15 h**

This step includes the lysis, reduction, alkylation, and cleanup of the samples, and trypsin addition performed by the robot. The user only moves the plate to the thermomixer for overnight digestion.

18. Place the sample plate without the lid in position 9.
19. Press “Check values and Run P2P” (Figure 3F).

***Note:*** The robot will take around 1 h and 57 min to perform lysis, reduction, alkylation, binding of the proteins to the beads, wash steps, and resuspension in ABC buffer with trypsin.

20. Seal the plate with sealing tape for 96-well plates in aluminum.

**CRITICAL:** Make sure the wells are properly sealed to avoid evaporation of the ABC buffer. (Troubleshooting 5).

21. Incubate for 15 h at 37°C at 1000 rpm on the thermomixer with the plate adapter and heated lid.

***Note:*** The thermomixer can be programmed to switch to 8°C after 15 h at 37°C.

***Note:*** If the flat-bottom plate adapter is not available, the plate can be fixed to a 96- or 384-well PCR plate adapter.

**CRITICAL:** The heated lid is necessary to avoid condensation and sample loss.

### Sample acidification and recovery

**Timing: 15 min**

This step consists of the acidification and recovery of the peptides.

***Note:*** If the robot was switched off, repeat steps 1–3 of the robot preparation.

22. Unselect everything in the input parameter tab.
23. Select “Perform acidification and recovery”.
  a. Set TFA value to 5 μL.
  b. Set the volume for transfer to 45 μL.
24. Place a new V-bottom plate in position 8.
25. Place a new box of tips in position 6.
26. In the labware, reagent and tips tab, check that the tips box location 3 is set according to the tips in position 3.

***Note:*** For this step, only 1 column of tips is needed in position 3.

27. Press “Calculate volumes” and “Update layout”.
28. Add 4 ml of 5% TFA in the sixth column of the solvent plate in position 8.
29. Place the sample plate without the seal in position 9.
30. Press “Check values and Run P2P”.

***Note:*** The robot will take 15 min for acidification and recovery. After this step, the beads should then be removed; however, residual beads might still be present.

**Pause point:** The samples can be sealed and frozen at −80°C.

### Transfer to the autosampler plate

**Timing: 5 min**

This step allows the automated transfer of 5 μl to the autosampler plate.

31. Remove the culture plate from the robot.
32. Add an autosampler V-bottom plate to position 8.
33. Make sure that the plate containing the samples is in position 9.
34. Unselect everything in the input parameter tab.
35. Select “Perform transfer to autosampler plate”.
36. Press “Check values and Run P2P”.

***Note:*** The robot will take 5 min to transfer the samples to a new plate. Afterwards, there should be 5 μl in each well.

37. The leftover samples can be sealed or transferred to PCR strips and stored at −80°C.

***Note:*** To avoid any residual beads, the leftover samples can be transferred to the PCR strips while keeping the plate on the magnet.

38. The autosampler plate can be loaded onto the LC system for MS analysis.

***Note:*** The peptide concentration can be determined by peptide BCA following the manufacturer’s instructions (Pierce™ Quantitative Peptide Assays & Standards Peptide Assay).

## Expected outcomes

We described a workflow for the preparation of MS samples from 5500 adherent cells in a 96-well format without the need for early transfer. The expected number of proteins identified depends on the acquisition method and instrument chosen. With the latest generation of instruments, such as the Orbitrap Astral, we anticipate the reproducible identification of more than 8000 proteins from 5500 cells.

The application of this workflow yields highly reproducible proteomic datasets across batches with a Pearson’s coefficient above 0.95 (Figure 4A), and a log2-transformed protein intensity distribution comparable across samples (Figure 4B). Data completeness analysis reveals a low proportion of missing values at the precursor and protein group level (Figures 4C and 4D).

**Figure 4:**
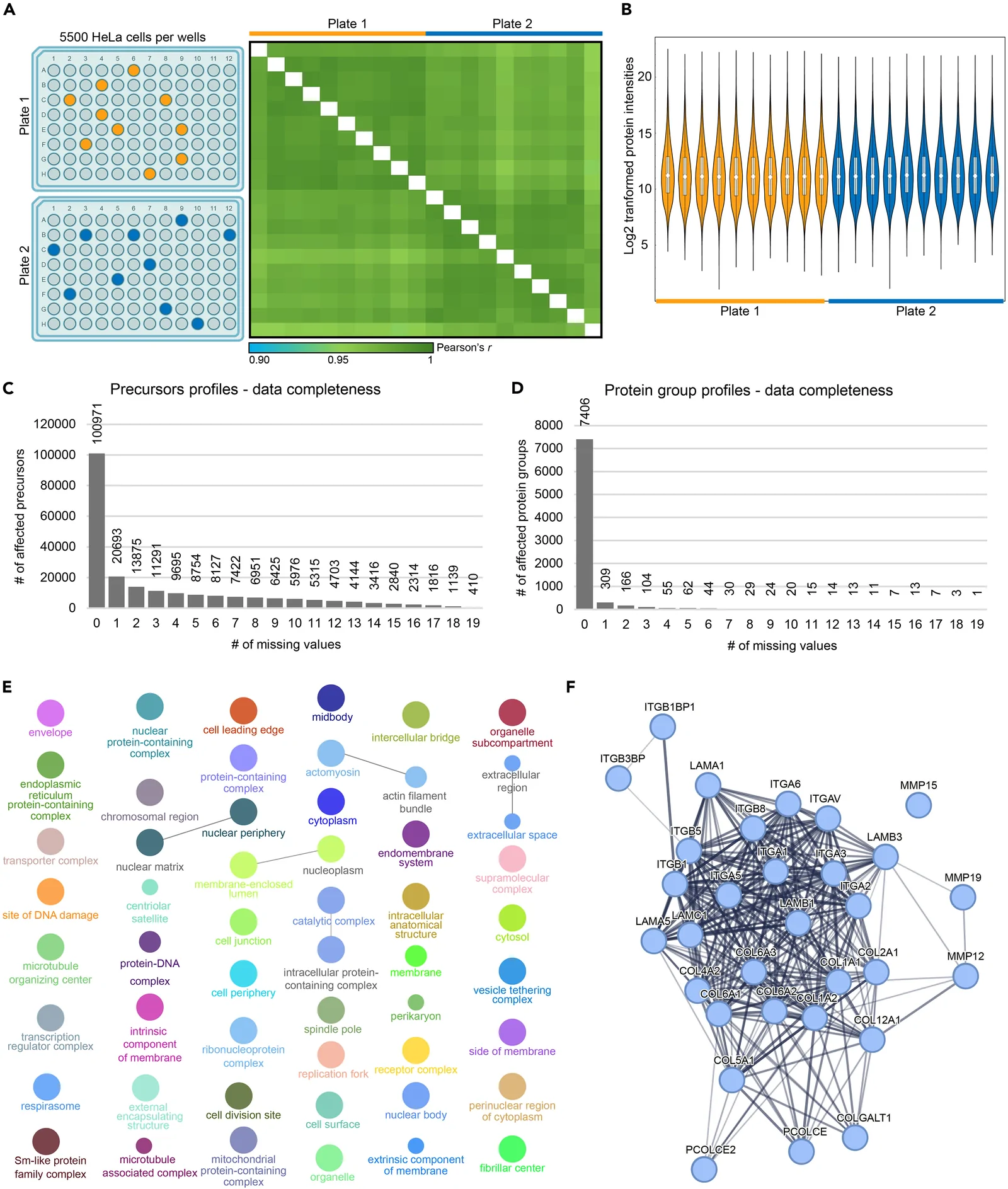
Sample preparation done with P2P is reproducible (A) Two plates have been processed on two different days. Ten samples per plate were randomly selected, and 3 μl were injected (around 200 ng). Pearson correlation between the samples shows reproducibility intra- and interplate. (B) Distribution of Log2-transformed protein intensities across samples. (C and D) Precursors and protein groups with missing values across the 20 samples from 2 plates. (E) Gene ontology (GO) cell component analysis of 7,288 commonly identified proteins isolated from 20 samples from 2 different batches. GO terms indicate the broad coverage of the proteome. Edges between the GO terms correspond to the term-term interrelation (kappa score on shared genes between terms). Results were created and visualized with ClueGO v2.5.10.^11^ (F) ECM proteins such as collagens, laminins, and integrins, along with associated enzymes were selected and visualized to illustrate the presence of key ECM components and associated interaction partners. Results were visualized with the STRING database.^12^

GO term analysis on the identified proteins shows the broad coverage of the diverse cellular components, indicating the ability to cover a representative part of the proteome with this workflow (Figures 4E and 4F).

To demonstrate the applicability of the workflow to detect biologically relevant changes, we performed a dose- and time-dependent treatment experiment with bortezomib, a clinically relevant proteasome inhibitor,^13^ which yielded clear alterations in the protein abundances (Figures 5 and 6).

**Figure 5:**
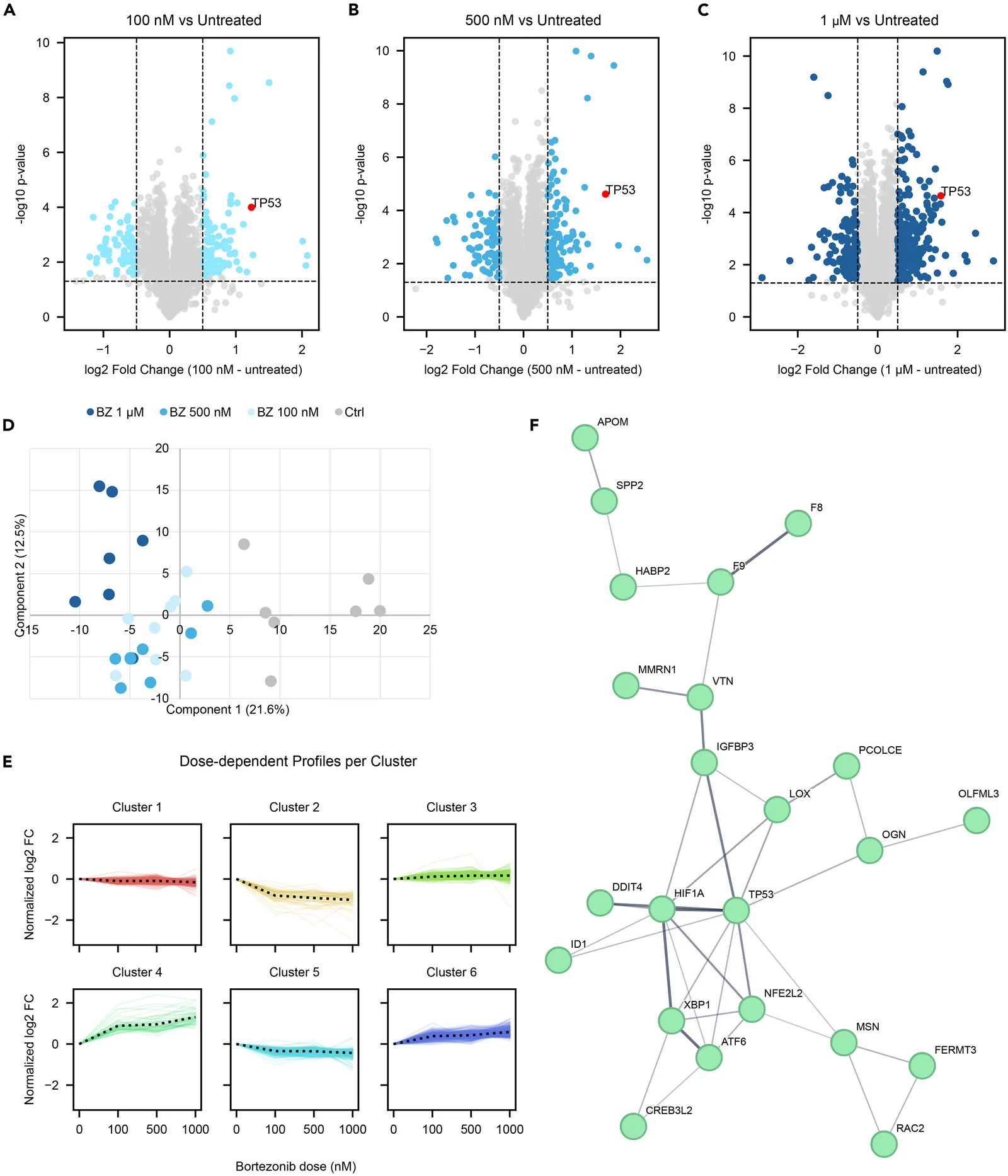
Dose-dependent proteome remodeling and accumulation of p53 upon bortezomib treatment (A–C) Volcano plots showing significantly regulated proteins compared to untreated cells after 2 h treatment with increasing concentration of bortezomib. Significant proteins are highlighted in blue (Benjamini-Hochberg adjusted p-value < 0.05 and |Log2 Fold Change|> 0.5), while p53 is highlighted in red. The volcano plots for A. 100 nM (160 highlighted proteins), B. 500 nM (211 highlighted proteins), and C. 1 μM (246 highlighted proteins) show that higher bortezomib concentrations induce progressively more significant protein regulation. (D) Principal component analysis (PCA) of 7949 commonly identified proteins, showing separation of samples according to treatment conditions. (E) Per-cluster panels of protein log2 fold-change trajectories across doses; K-means clusters (k=6). Proteins were filtered by one-way ANOVA across timepoints (Benjamini–Hochberg FDR q < 0.05) prior to clustering. Thin lines: individual proteins; dotted black line: cluster mean. (F) STRING databasenetwork of proteins from cluster 4, showing p53 and similarly dose-dependent upregulated proteins.^12^ Edge thickness represents the strength of data supporting a biological association (minimum confidence score: 0.400).

**Figure 6:**
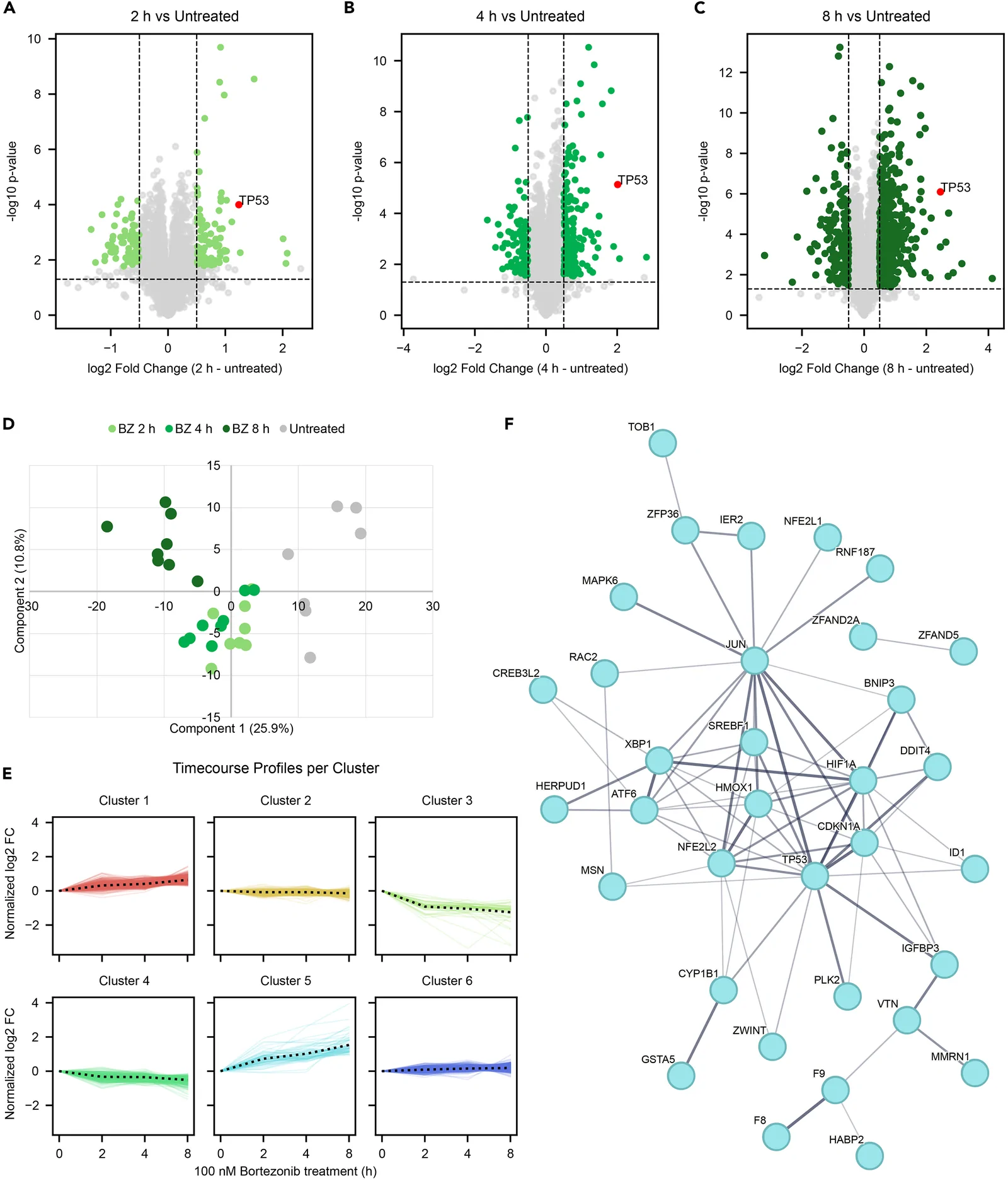
Time-dependent proteome remodeling and accumulation of p53 upon bortezomib treatment (A–C) Volcano plots showing significantly regulated proteins compared to untreated cells after 100 nM bortezomib treatment with increasing incubation time. Significant proteins are highlighted in green (Benjamini-Hochberg adjusted p-value < 0.05 and |Log2 Fold Change|> 0.5), while p53 is highlighted in red. The volcano plots for A. 2 h (153 highlighted proteins), B. 4 h (254 highlighted proteins), and C. 8 h (554 highlighted proteins) show that longer incubation times induce progressively more significant protein regulation. (D) Principal component analysis (PCA) of 8017 commonly identified proteins, showing separation of samples according to treatment conditions. (E) Per-cluster panels of protein log2 fold-change trajectories across timepoints; K-means clusters (k=6). Proteins were filtered by one-way ANOVA across timepoints (Benjamini–Hochberg FDR q < 0.05) prior to clustering. Thin lines: individual proteins; dotted black line: cluster mean. (F) STRING databasenetwork of proteins from cluster 5, showing p53 and similarly time-dependent upregulated proteins.^12^ Edge thickness represents the strength of data supporting a biological association (minimum confidence score: 0.400).

In the dose-response evaluation (Figure 5), higher bortezomib concentrations induce progressively more significant changes in protein abundances, including the known accumulation of p53,^13^ showing that the workflow allows the study of dose-dependent proteome remodeling. Similarly, the time-course (Figure 6) reveals that increasing exposure to the lower dose of bortezomib leads to larger numbers of regulated proteins, with p53 also accumulating over time.

Together, these results demonstrate that the workflow enables robust and reproducible quantification of proteome changes and provides confidence that it can be applied to other perturbation studies.

## Limitations

This workflow is based on a specific setup of the Agilent Bravo Liquid Handling Platform. With a different setup, the user might have to contact the Agilent Automation team for support. In case of cells yielding very low amounts of proteins, the generated data might vary more. A solution could be to increase the number of seeded cells per well.

## Troubleshooting

### Problem 1

Uneven cell distribution reduces reproducibility.

### Potential solution

Gently resuspend the cell suspension before dispensing it in the wells and mix regularly to prevent cells from settling.

### Problem 2

Beads are not delivered in every well (Figure 7).

**Figure 7:**
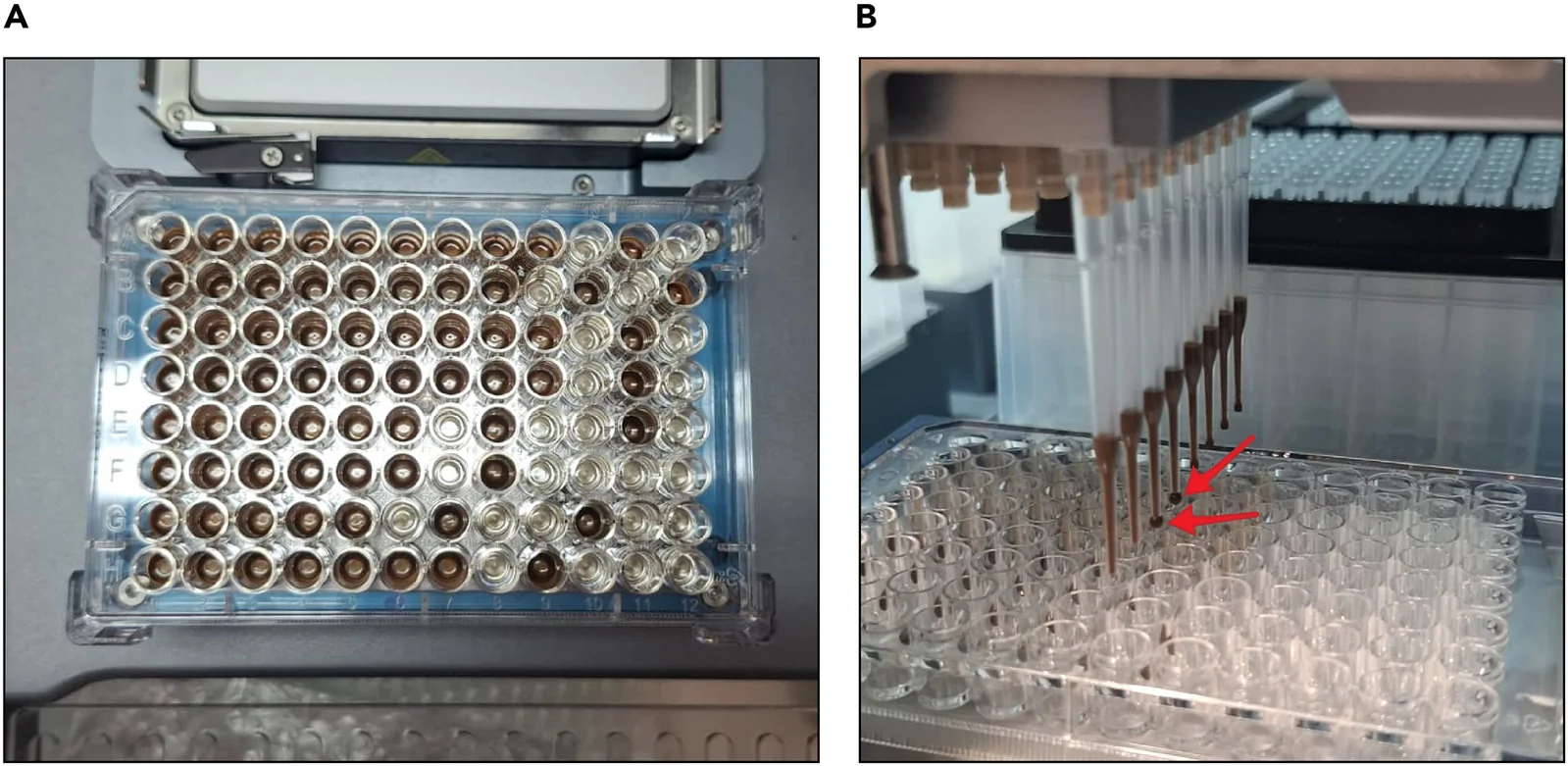
Failure in beads delivery due to incorrect labware settings (A) The beads are not delivered to some of the wells. (B) Arrows show the drops of beads that were not delivered in the wells due to failure of “tip touch” of the wall.

### Potential solution

The tips might not “tip touch” the wall of the well; adjust the “well diameter” and the “row-wise well to well” settings in the labware editor.

### Problem 3

After pressing “Check values and Run P2P”, the protocol does not start, and a sound is produced.

### Potential solution

There might be an error in the calculated values or in the retrieval of the labware. Press “Retrieve data from labware DB and calculate max volume”, “Calculate volumes” and “Update layout” again.

Another possible cause is the missing labware in the Labware Editor. Make sure the name in the form and in the Labware Editor correspond.

### Problem 4

The beads are not homogeneously delivered across rows or columns (Figure 8).

**Figure 8:**
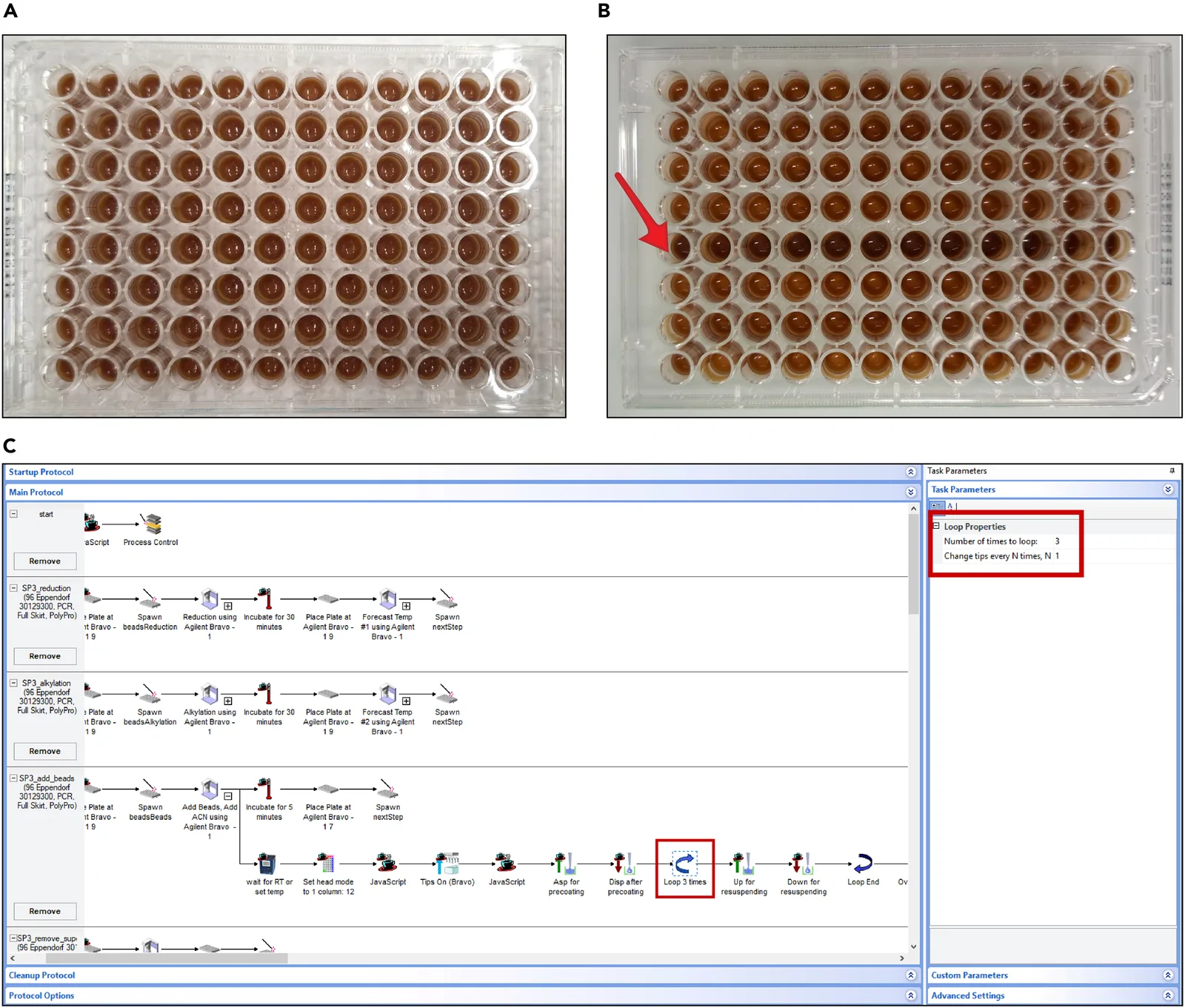
Unequal beads delivery due to failed resuspension (A) Homogeneous amount of beads delivered across the whole plate. (B) One row (red arrow) presents more beads due to failed resuspension of the beads before delivery to the reagent plate. The beads should be carefully resuspended before adding them to the reagent plate. (C) In case of unequal beads across columns, the number of loops in the protocol can be increased to improve the resuspension.

### Potential solution

For rows: Different concentrations of beads were delivered on the reagent plate. To avoid this, carefully resuspend the beads before adding them to the reagent plate.

For columns: Beads potentially precipitated, and the resuspension step was not sufficient. Increase the number of loops in the protocol to allow a good resuspension before delivering the beads to the wells.

### Problem 5

Some of the samples were not recovered properly. There is no/less volume recovered.

### Potential solution

The sample evaporated during incubation at 37°C. To avoid it, make sure to seal each well properly before the incubation. If there was no volume, add 30 μl of ABC buffer to the beads, shake the plate quickly, and recover by hand with the help of the magnet.

## Resource availability

### Lead contact

Requests for further information and resources should be directed to and will be fulfilled by the lead contact, Prof. Joern Dengjel (joern.dengjel@unifr.ch).

### Technical contact

Technical questions on executing this protocol should be directed to and will be answered by the technical contact, Sibilla Sander (sibilla.sander@unifr.ch).

### Materials availability

The workflow files for the Bravo automated liquid handling platform can be obtained from Agilent free of charge.

### Data and code availability

The MS proteomics data have been deposited to the ProteomeXchange Consortium via the PRIDE^14^ partner repository with the dataset identifier PRIDE: PXD082640.

## Acknowledgments

This work was supported by the University and the Canton of Fribourg as part of the SKINTEGRITY.CH research network and by the 10.13039/501100001711Swiss National Science Foundation with NRP79 (grants 407940_206394 and 316030_229588). We thank the Agilent automation team for sharing the original workflow with us.

## Author contributions

Conceptualization, S.S., M.S., S.B., and J.D.; methodology, S.S.; validation, S.S. and S.B.; formal analysis, S.S. and S.B.; investigation, S.S. and S.B.; writing – original draft, S.S. and J.D.; writing – review and editing, S.S., S.B., M.S., and J.D.; visualization, S.S.; supervision, J.D.; project administration, J.D.; funding acquisition, J.D.

## Declaration of interests

The authors declare no competing interests.
